# The Efficacy of Ketogenic Diet and Associated Hypoglycemia as an Adjuvant Therapy for High-Grade Gliomas: A Review of the Literature

**DOI:** 10.7759/cureus.251

**Published:** 2015-02-27

**Authors:** Kunal Varshneya, Christine Carico, Alicia Ortega, Chirag G. Patil

**Affiliations:** 1 Center for Neurosurgical Outcomes Research, Maxine Dunitz Neurosurgical Institute, Cedars-Sinai Medical Center; 2 Cedars-Sinai Medical Center

**Keywords:** high-grade glioma, ketogenic diet, hypoglycemia, neurosurgery, hyperglycemia, glioblastoma

## Abstract

Background: A high-fat, low-carbohydrate diet, often referred to as a ketogenic diet (KD), has been suggested to reduce frequency and severity of chronic pediatric and adult seizures. A hypoglycemic state, perpetuated by administration of a KD, has been hypothesized as a potential aid to the current standard treatments of high-grade gliomas.

Methods: To understand the effectiveness of the ketogenic diet as a therapy for malignant gliomas, studies analyzing components of a KD were reviewed. Both preclinical and clinical studies were included. The keywords “ketogenic diet, GBM, malignant glioma, hyperglycemia, hypoglycemia” were utilized to search for both abstracts and full articles in English. Overall, 39 articles were found and included in this review.

Results: Studies in animal models showed that a KD is able to control tumor growth and increase overall survival. Other pre-clinical studies have suggested that a KD sustains an environment in which tumors respond better to standard treatments, such as chemoradiation. In human cohorts, the KD was well tolerated. Quality of life was improved, compared to a standard, non-calorie or carbohydrate restricted diet. Hyperglycemia was independently associated with diminished survival.

Conclusion: Recent clinical findings have demonstrated that induced hypoglycemia and ketogenic diet are tolerable and can potentially be an adjuvant to standard treatments, such as surgery and chemoradiation. Other findings have advocated for KD as a malignant cell growth inhibitor, and indicate that further studies analyzing larger cohorts of GBM patients treated with a KD are needed to determine the breadth of impact a KD can have on GBM treatment.

## Introduction and background

Glioblastoma (GBM) is the most prevalent and aggressive primary intracranial tumor. Although relatively rare, with an occurrence rate of 2 out of 100,000 people, GBMs account for over 82% of all malignant gliomas [1]. Despite multimodal treatment, including radiation and chemotherapy following aggressive surgical resection, the prognosis is still poor with a median survival of 13-15 months post-diagnosis [2-3]. Similar to GBMs, traditional treatment of anaplastic astrocytomas (AAs) includes primary resection followed by radiation and chemotherapy [4-5]. Additionally, given that GBMs and AAs are often located in the eloquent cortex and have innate abilities to proliferate extensively, recurrence often occurs approximately 32-36 weeks after initial treatment [1]. Radiation and chemotherapy have been shown to increase progression-free survival (PFS); however, overall survival (OS) for GBM patients remains poor with only 15-20% of patients surviving longer than three years [6]. Recent basic laboratory studies have demonstrated that alternative treatments, such as progesterone therapy and restrictive diets, may inhibit angiogenic molecular pathways in glial cells, attenuating malignant glioma growth [7-10].

A high-fat, low-carbohydrate diet, often referred to as a ketogenic diet (KD), has been suggested to reduce frequency and severity of chronic pediatric and adult seizures [11-14]. Recent studies have demonstrated that a calorically-restricted diet administered to mice infused with a malignant mouse astrocytoma (CT-2A) or a human glioma (U87-MG) was effective in decreasing vascularity, increasing programmed cell death, and was associated with diminished levels of insulin-like growth factors [15]. The effectiveness of calorie restriction or a ketogenic diet can be understood through analysis of its biochemistry. A landmark study by Nobel laureate Otto Warburg demonstrated neoplastic metabolic dependence on aerobic glycolysis for energy production [16]. A consumed fat molecule (triacylglycerol) is catabolized into free fatty acids and eventually Acetyl-CoA. If increased fatty acid oxidation elevates the levels of Acetyl-CoA to surpass the capacity of the citric acid cycle, excessive levels of ketone bodies accumulate (β-hydroxybutyrate (BHB) and acetoacetate (ACA)) [8, 17-18]. Normal neurons and glial cells are able to metabolize BHB and ACA; however, neoplastic cells appear less able to utilize these sources for energy derivation. Cancer (CP1) cells also have markedly elevated levels of reactive oxygen species (ROS) that have been associated with angiogenesis and cell proliferation through mediation of vascular endothelial growth factor (VEGF) and hypoxia inducible factor 1 (HIF-1). Ketone bodies have been linked to ROS reduction *in vivo *[19]*. *Due to tumor cells’ metabolic dependence on glucose and glutamate, elevated levels of ketone bodies in the intracranial region diminish glycolytic levels and inhibit angiogenesis [7, 17, 20-21].

## Review

### Methods

To understand the effectiveness of the ketogenic diet as a therapy for malignant gliomas, studies analyzing components of a KD and its implementation in both preclinical and clinical studies were reviewed. The focus of this review was on the anti-tumor effects of a KD in high-grade gliomas. A literature review was conducted and articles from 1976 to 2014 in PubMed were reviewed for their relation to efficacy of the ketogenic diet as alternative therapy for glioma. Both preclinical and clinical studies were included. The keywords “ketogenic diet,” “GBM,” “malignant glioma,” “hyperglycemia,” and “hypoglycemia” were utilized to search for both abstracts and full articles in English. Overall, 39 reviews, preclinical studies and clinical studies were collected for use in this review. Fifteen of these studies were explicitly discussed in this review whereas the remaining articles were simply referenced. Two clinical trials currently being conducted were also described.

### Discussion

Preclinical Studies and Carbohydrate Restriction

Cachexia, also known as wasting syndrome, is frequently reported in cancer patients. In response, regulatory starvation mechanisms promote plasma ketone body levels, which have been observed to reduce rates of glycolysis and gluconeogenesis [22]. It has been suggested that cancerous cells have an intrinsic dependence on glycolysis. The induction of ketosis (the production of ketone bodies), may reduce malignant cell growth [23-26]. In a 1979 study, Magee, et al. studied 36 C57BL/6 mice in two different environments; one in which half the mice were fed water and sucrose (Group 1), and another where mice were only fed water and polyunsaturated, blended vegetable oil (Group 2). All mice had melanoma cells implanted in their tails, and after two weeks of incubation in their respective environments, two independent observers determined malignant cell growth. This study demonstrated that an increased saturation of BHB, which was observed in the oil-fed mice, hindered the proliferation of malignant cells in culture. The lack of available glucose in Group 2 induced a hypoglycemic state. It was inferred that since ketone bodies were unable to be metabolized, malignant cell growth was greatly dampened. Given the effect of ketone bodies on generalized malignant cell growth, a potential link between this carbohydrate-fasted state and cancer treatment was postulated [19].

Critics of the previous study argued that perhaps a lack of carbohydrates in the diet did not contribute to the inhibition of malignant cell growth, but instead this inhibition was due to fewer dietary calories administered throughout the study. A 2003 study by Seyfried, et al. attempted to determine the effects of calorie restriction on malignant brain cancers while controlling for carbohydrate restriction. Mice were implanted with a CT-2A malignant astrocytoma and placed in four treatment categories: a standard, unrestricted calorie diet (SD-UR); a ketogenic, unrestricted calorie diet (KD-UR); a standard diet, restricted to 40% of the SD-UR calories (SD-R); or a ketogenic diet, restricted to 40% of the SD-UR (KD-R). Body weights, tumor weights, and BHB concentrations were measured two weeks after implantation. Tumor growth was rapid in both of the unrestricted diet groups (SD-UR and KD-UR). However, in the restricted diet cohorts, malignant growth was significantly impaired. Overall, an 80% reduction in tumor growth was correlated with caloric restriction, with little deviation arriving from the origin of calories [27]. This study suggested that the amount of calories and not necessarily their origin was more integral for malignant growth. However, further studies had yet to establish a calorically restricted ketogenic diet as a potential aid in the management of cancer. 

**Figure 1:**
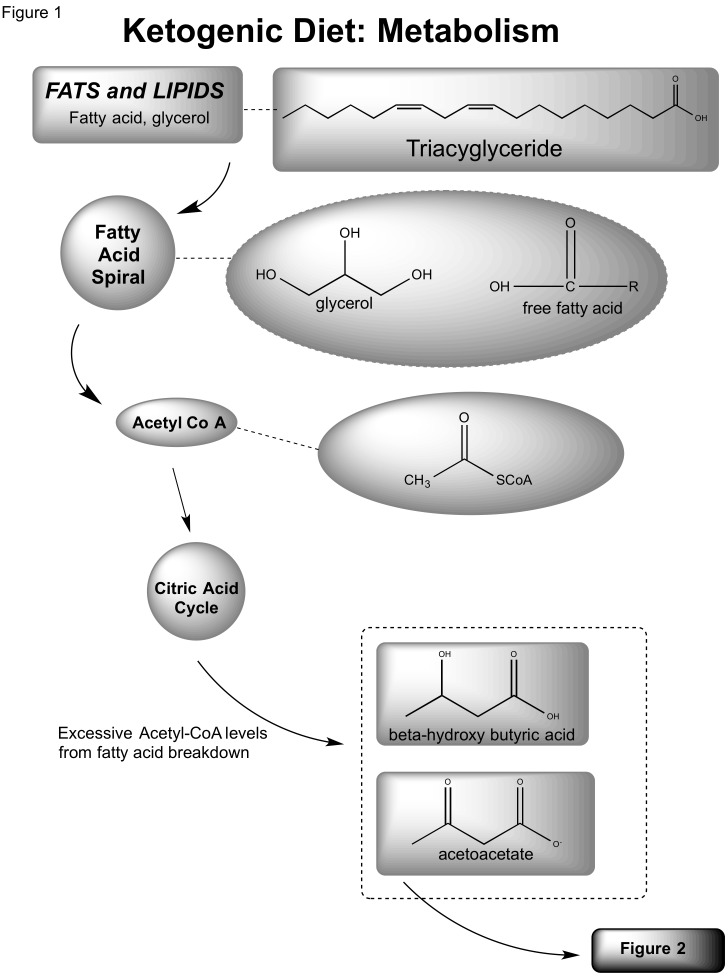
Ketogenic diet: metabolism

**Figure 2:**
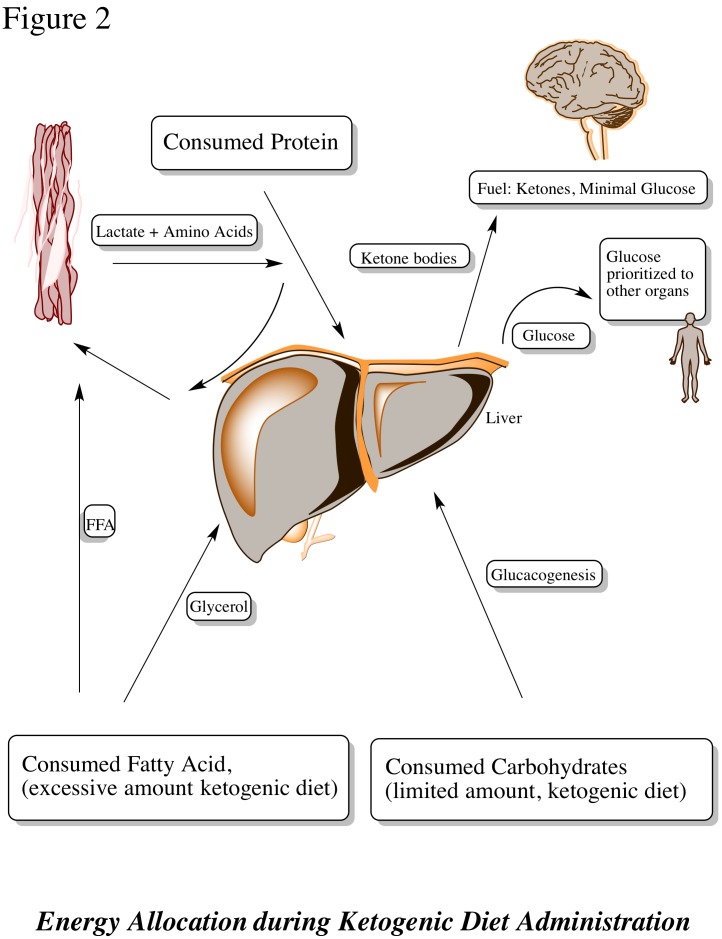
Energy allocation during ketogenic diet administration

Zhou, et al. recently introduced KetoCal®, a ketogenic diet that is no different from previously discussed KDs except that KetoCal® had become the standardized KD treatment for epileptic patients. The KetoCal® diet was investigated as a potential therapy to minimize the vascularity and malignancy of intracranial neoplasms, specifically astrocytomas [24]. In their study, mice were categorized into calorically unrestricted and restricted KetoCal® diets. This cohort was compared to a control group receiving a standard, high-carbohydrate/low-fat diet. Whereas both the unrestricted and restricted KetoCal® diets slowed tumor growth, decreased vascularity, and increased mouse survival relative to the control group, the restricted KetoCal® diet was associated with the best survival overall. A variety of metabolism-related metrics were measured. Precursors, such as plasma glucose level and glutamate breakdown, were significantly lowered in the restricted KetoCal® diet group versus the restricted standard, high-carbohydrate diet group (Figures 1, 2). On the molecular biological scale, gene expression for mitochondrial enzymes, such as BHB dehydrogenase and succinyl-CoA: 3-ketoacid CoA transferase, proteins that stimulate metabolism and growth, had diminished. Tumor density had also decreased significantly. This implied that the abundance of ketone bodies were effective in slowing brain metabolism, specifically in neoplastic cells. Marsh, et al. later studied the effects of a metabolism-inhibiting drug in conjunction with the KD on malignant gliomas [28]. The glucose molecule exposed to a hydroxyl group, known as 2-deoxy-d-glucose (2-DG), is a competitive inhibitor of glucose in glycolysis. Low-dose 2-DG (25 mg/kg) has been shown to produce similar calorie and energy restrictions. A restricted ketogenic diet (KD-R) and 2-DG was implemented in mice implanted with malignant gliomas and the results were compared with the control groups of a SD-UR and KD-R with and without 2-DG administration. After treatment, tumor weights were significantly lower in the KD-R and the KD-R+2-DG versus the control cohorts: 48% and 80%, respectively. Overall tumor size was smallest in the KD-R+2-DG, implying that a simultaneous KD-R+2-DG therapy may have potential to shrink tumors [28].

Studies have shown that a KD-R can be a beneficial adjuvant treatment for malignant astrocytomas; however, few studies have been able to demonstrate a better prognosis in GBM cohorts. In 2010, Stafford, et al. induced a hypoglycemic state in mice and attempted to determine the effects on a GL261 mouse glioma, an experimental model of a GBM. One cohort of mice was fed a KD-UR while another received an SD-UR. After the incubation period passed, the KD-UR cohort was correlated with better survival and slower neoplastic proliferation rate. Similar to Zhou, et al., the KD-UR treatment was associated with diminished metabolism and tumor growth on a molecular biological scale. Diminished amounts of mitochondrial enzymes and low levels of reactive oxygen species (ROS), compounds associated with metabolism promotion, were observed implying that a KD-UR may inhibit glucose metabolism via inhibition of glycolysis. Genes that are expressed in elevated levels during metabolism were also affected. Genes encoding for enzymes commonly present in saturated levels during glycolysis, such as cyclooxygenase 2, glutathione peroxidases 3 and 7, and peroxiredoxin 4, were sparse. This was another indication that a KD-UR could potentially stagnate tumor growth by inhibiting metabolic components. In addition to increased survival rates among the KD-UR mice, these observations suggest that a KD-UR, although not calorically limited, may still aid in the treatment of intracranial neoplasms [29]. In 2011, Maurer, et al. used a similar method of classification of mouse cohorts and were able to observe elevated levels of 3-hydroxybutyrate dehydrogenase 1 and 2 (BDH1 and 2), 3-oxoacid-CoA transferase 1 (OXCT1), and acetyl-CoA acetyltransferase 1 (ACAT1) — common ketone body metabolizing agents. Counterpart glycolytic agents were absent and glioblastoma growth stagnated. Plasma glucose levels remained low and overall results were consistent with previous studies on the ketogenic diet, supporting the efficacy of a KD-UR in malignant intracranial tumor treatment [30]. A final implementation of the popular pediatric epilepsy treatment, KetoCal®, was studied in conjunction with a radiation (2 x 4 Gy) treatment in malignant gliomas. As expected, BHB levels were elevated in the KetoCal® rat cohort (p = 0.0173) and the bioluminescent marker reacting with the tumor cells in the rats fell below detection (p < 0.0001). Furthermore, mean survival in this group was five days longer than in the control rats receiving a standard diet. These results suggested that the ketogenic diet significantly enhanced the anti-tumor effects of radiation treatment [24, 27-30]. These studies portrayed the efficacy of the ketogenic diet as a potential adjuvant therapy in addition to traditional treatment of GBMs, effectively building the foundation for clinical trials.

Clinical Effectiveness of a KD in Anaplastic Astrocytoma

Although the ketogenic diet has been a standard treatment for epileptic patients for decades, only recently has the KD been utilized in human subjects with cancer. Given the significant results of tumor shrinkage and improved survival in mouse models, it was hypothesized that a KD-UR may have a survival impact on cerebral cancer patients [31]. In 1995, Nebeling, et al. induced ketosis in pediatric astrocytoma patients by feeding them a KD consisting of 60% medium-chain triglyceride (MCT) oil, 20% protein, 10% carbohydrate, and 10% dietary fats. In an attempt to emulate the results of previous preclinical studies, tumor growth, glucose uptake, plasma glucose levels, and ketone body levels were monitored. After seven days of KD initiation, plasma ketone body levels increased 20-fold and blood glucose levels dropped significantly. Through PET scan analysis, the tumors were also observed to have reduced glucose uptake by 21.8%. This pioneer study concluded that a ketogenic diet was safe for humans, and in fact supported previous findings, and stated that a KD could potentially supplement cancer treatment [32]. Since this study did not have access to long-term patient data, a significant limitation was the omission of progression-free and overall survival data of patients treated with a KD. However, given the KD’s previously observed effectiveness, later studies sought to determine the outcome of patients initially treated with a KD.

Clinical Presence of Elevated Levels of Serum-Glucose and GBM

In 2008, a retrospective study by McGirt, et al. was conducted analyzing a hyperglycemic state and its effect on the prognosis of GBM patients [33]. A hyperglycemic state is the opposite of what a KD perpetuates, and through isolating the effects of glucose levels on the tumor, it was determined that chronic hyperglycemic states are independently associated with diminished survival following a resection, regardless of steroid usage. A cohort of 297 GBM patients who had undergone surgical tumor resection was studied. Persistent hyperglycemia was defined by at least three observations of plasma-glucose levels surpassing 180 mg/dl within one to three months postoperatively. Variables, such as older age (p = 0.001), lower Karnofsky Performance Scale score (p=0.001), higher tumor grade (p=0.001), primary versus secondary resection (p=0.008), temozolomide administration (p=0.007), and continued outpatient dexamethasone therapy in addition to elevated levels of hyperglycemia were associated with diminished overall survival. Following initial GBM resection, hyperglycemic patients were observed to have a significantly reduced survival (5 vs. 11 months, p < 0.05). This observation was consistent with the secondary resection cohort, which also demonstrated a reduced survival in hyperglycemic patients (5 vs. 9 months, p < 0.05). Overall, even GBM patients without recurrent tumors experienced a significantly worse prognosis with high glucose levels (8 vs. 13 months, p < 0.05) [31]. In a similar study by Derr, et al., 191 newly diagnosed GBM patients were divided into four cohorts of mean plasma glucose levels according to measurements taken weekly during radiation treatment. The four quartiles of plasma glucose in these patients were defined as follows: quartile one (< 94 mg/dL), quartile two (94 to 109 mg/dL), quartile three (110 to 137 mg/dL) and quartile four (> 137 mg/dL). Median survival among these groups was 14.5, 11.6, 11.6, and 9.1 months, respectively. After adjusting for intergroup differences, such as age, extent of resection, and adjuvant therapies, significantly reduced survival in patients with increased levels of plasma glucose was observed (p = 0.041) [34].

Another experiment to test the effects on hyperglycemia on lower grade gliomas with a cohort of 189 patients revealed a correlation between elevated plasma glucose levels and increased malignancy and likelihood of recurrence, with diminished survival [35]. These studies demonstrated that elevated glucose levels may be detrimental to overall survival, indicating that a treatment that may inhibit this may have potential in managing malignant growth. Further studies inferred that since preventing hyperglycemia may aid in the treatment of low-grade gliomas, this practice might also have a place in the treatment of GBMs.

Clinical Studies of the KD in Glioblastoma Patients

A case report in 2010 discussed a 65-year-old female being admitted with symptoms of memory loss, chronic headaches and nausea. With a family history of cancer and the given symptoms, she was diagnosed with a multi-centric GBM tumor. A KD-R was administered. Following the KD-R, treatment of surgery, radiation and chemotherapy was given. After two months of treatment, a MRI scan revealed that no residual tumor cells were present [36]. This success was rare and sparked the interest of many in the KD-UR, as it was the only novel aspect of the treatment. Following this report, a pilot study was conducted in 16 patients presenting with advanced stage lung, liver or bone cancers. A KD was administered to these patients, but due to a variety of reasons, such as an unpalatable nature of KD and death, only five subjects were able to complete the three-month intervention. These patients reported less insomnia and improved overall mood, two important quality of life parameters [37]. Another study of the KD and its efficacy as a glioblastoma treatment was conducted by Champ, et al. in which GBM patients were retrospectively analyzed from August 2010 to April 2013. Serum glucose and plasma ketone levels were monitored along with overall toxicity (Common Terminology Criteria for Adverse Events Grades 1-5; 1 being mild and 5 being death) and dexamethasone dosage. Six of 53 patients diagnosed with a GBM were fed a KD-UR while the other 47 were fed a standard, carbohydrate-rich diet. After monitoring the patients for 14 months, four of the six KD-R patients were still alive with no signs of chronic hypoglycemia, fatigue, or other potential side-effects. Mean plasma glucose levels were measured, and the standard diet (SD) patients were found to have measurements of 122 mg/dl versus 84 mg/dl in the ketogenic diet fed patients. Serum levels were monitored and glucose levels were found to have dropped precipitously whereas ketone body presence rose. The KD-UR was well tolerated throughout the study with nominal side effects, such as weight loss (potentially due to a 30% reduction of calories), and Grades I and II fatigue. KD-R cohort recurrence was observed at a median time of 10.3 months post-diagnosis. Most patients responded well to the KD-UR, with one patient recurring at seven months and another not showing any signs of recurrence 12 months after initial treatment. One of the other four patients died at six months due to a more aggressive, multifocal GBM. Overall, the KD was well tolerated and reduced serum glucose levels significantly without any reported symptoms of chronic hypoglycemia. It was suggested that since a hypoglycemic state is detrimental to tumor growth, KD could be an option prior to more standard treatments, such as surgery and chemoradiation [38]. 

Many studies have suggested that elevated plasma glucose levels are correlated with accelerated tumor growth and poor progression-free and overall survival. Some have demonstrated that a KD and KD-UR are both effective in aiding the management of malignant growth, where a KD-UR had the highest efficacy [28, 31, 33, 39]. The success of these studies prompted further research into the aspects of the KD that made it effective, such as an induced hypoglycemic state and the inhibition of glutamate to glutamine conversion [38]. Hyperglycemia, which is inhibited by a KD, was shown to amplify systematic symptoms and increase tumor density, hindering prognosis significantly [32, 35]. These results promoted the KD as a potential supplement to the standard treatment of GBM; however, the translation to human subjects was unknown. Later clinical studies demonstrated that the KD is a safe and tolerable adjuvant therapy [38].

At the time of this review, there are two ongoing clinical trials set to be completed by 2016 studying the efficacy of a KD in GBM patients: a Phase I/II and a Phase II trial. In the Phase I/II trial, GBM patients will be administered a traditional 4:1 ratio of fat to carbohydrate KD while being treated chemoradiation for six weeks. Monthly chemotherapy will be given afterwards. Patients will have an initial MRI scan, weekly blood tests to monitor ketone levels and a final MRI test [40]. In the Phase II trial, recurrent GBM will be treated with a similar KD as the previous trial, along with chemoradiation. Following the treatment, bevacizumab may be administered [41]. Both studies aim to determine the tolerability and safety of a KD, along with its efficacy as a tumor-shrinking agent. Preclinical and clinical studies have both been able to suggest the effectiveness and tolerability of the KD and the KD-UR; however, the precise success of tumor shrinkage exhibited in the preclinical trials has not been explicitly carried over into the clinic. It is difficult to draw conclusions based on these studies given their lack of large cohorts—the largest being a group of six patients being treated with a KD [38]. Moreover, the exact scientific mechanisms of how the KD works are still unclear. Studies dissecting the aspects of a KD and finding the exact attribute of a KD that provides benefits are warranted. Furthermore, larger cohort studies to show a more conclusive and statistically significant improvement of KD therapy on GBM patient prognosis are needed. Table 1 is a summary of the most significant studies implementing the ketogenic diet in glioma patients in the animal cohort. Table 2 analyzes the same variables in the human cohort. Both tables are organized based on cohort size and levels of evidence (I - VII).

**Table 1:** Animal studies of calorie restricted or ketogenic diet with diagnosis of glioma

Authors	Summary of Study	Level of Evidence	Cohort	Conclusions
Adelwahab MG, et al. [31]	Mice were implanted with malignant gliomas, given 2 x 4 Gy radiation and administered either a SD or a KD; tumor growth was monitored with in vivo imaging	1	11	Animals fed KD had elevated levels of β-hydroxybutyrate (p = 0.0173) and an increased median survival of approximately 5 days relative to animals maintained on SD. KD plus radiation treatment were more than additive, and in 9 of 11 irradiated animals maintained on KD, the bioluminescent signal from the tumor cells diminished below the level of detection (p<0.0001).
Marsh J, et al. [28]	Mice were implanted with malignant astrocytomas and administered SD or KD; within these groups, half were given 2-deoxy-D-glucose	1	11	Tumor weights were about 48% and 80% lower in the KD-R and in the KD-R+2-DG groups, respectively, than in the SD-UR group. Mouse health and vitality was better in the KD-R group than in the KD-R+2-DG group. Astrocytoma growth was reduced more in the KD-R mouse group supplemented with 2-DG than in the mouse groups receiving either dietary restriction or 2-DG alone, indicating a synergistic interaction between the drug and the diet.
Maurer GD, et al. [30]	Rats were implanted with a glioma and administered SD or KD	2	24	In vivo, the ketogenic diet led to a robust increase of blood 3-hydroxybutyrate, but did not alter blood glucose levels or improve survival.
Mukherjee P, et al. [15]	Mice were implanted with a glioma (U87-MG), an astrocytoma (CT-2A), or an ependymoma (EPEN) and were administered calorie-restricted diets	1	83	The weights of the CT-2A, EPEN, and U87-MG tumors were approximately 80%, 63%, and 60% less with a calorie-restricted diet than standard, caloric surplus diet.
Seyfried TN, et al. [27]	Mice were implanted with a glioma and were fed either a standard diet unrestricted (SD-UR), a ketogenic diet unrestricted (KD-UR), the SD restricted to 40% (SD-R), or the KD restricted to 40% of the control standard diet (KD-R).	1	33	CT-2A growth was rapid in both the SD-UR and KD-UR groups, but was significantly reduced in both the SD-R and KD-R groups by about 80%.
Zhou W, et al. [24]	Adult mice were implanted orthotopically with the malignant brain tumors and KetoCal was administered to the mice in either unrestricted amounts or in restricted amounts to reduce total caloric intake	1	34	KetoCal® administered in restricted amounts significantly decreased the intracerebral growth of the CT-2A and U87-MG tumors by about 65% and 35%, respectively. Tumor microvessel density was less in the calorically restricted KetoCal® groups than in the calorically unrestricted control groups. Moreover, gene expression for the mitochondrial enzymes, β-hydroxybutyrate dehydrogenase and succinyl-CoA: 3-ketoacid CoA transferase, was lower in the tumors than in the contralateral normal brain.

**Table 2:** Human studies of calorie-restricted or ketogenic diet with diagnosis of malignant glioma

Authors	Summary of Study	Level of Evidence	Cohort	Conclusions
McGirt M, et al. [33]	Retrospective analysis of patients with a diagnosis of a high-grade hemispheric glioma and persistent elevated levels of plasma glucose, defined by higher than 180 mg/dl at least three times postoperatively within 3 months of surgery and its association with survival was studied.	2	367	Persistent outpatient hyperglycemia (relative risk, 1.79; 95% confidence interval, 1.05-3.05, P = 0.03) remained independently associated with decreased survival. Median survival for persistently hyperglycemic versus normal-glycemic cohorts was 5 and 11 months, respectively.
Chaichana KL, et al. [35]	Retrospective analysis of patients with a diagnosis of a low-grade hemispheric glioma and persistent elevated levels of plasma glucose, defined by higher than 180 mg/dl at least three times postoperatively within 3 months of surgery and its association with survival was studied.	2	182	Five-year overall survival, progression-free survival and malignancy-free survival for persistent hyperglycemia versus relatively euglycemic cohorts were 43% versus 84%, 16% versus 46%, and 46% versus 77%, respectively.
Champ CE, et al. [38]	Retrospective analysis of patients between 2010-2013 who underwent KD therapy along with the standard treatment	2	53	The diet was well tolerated with no Grade III toxicity and one episode of Grade II fatigue. No episodes of symptomatic hypoglycemia were experienced. Four patients are alive at a median follow-up of 14 months. The mean blood glucose of patients on a standard diet was 122 versus 84 mg/dl for those on a KD.
Derr RL, et al. [34]	Retrospective analysis of patients with GBMs and their outcomes (overall survival) based on postoperative glycemic levels. Patients were divided into quartiles: quartile one (< 94 mg/dL), quartile two (94 to 109 mg/dL), quartile three (110 to 137 mg/dL), and quartile four (> 137 mg/dL).	2	191	Median survival times for patients in quartiles one, two, three, and four were 14.5, 11.6, 11.6, and 9.1 months, respectively.
Schmidt M, et al. [37]	Prospective study administering KD to patients with advanced metastatic cancer, hoping to determine feasibility and effectiveness of KD as an adjuvant treatment to standard of care.	1	16	Patients reported less insomnia and better overall mood; cholesterol and blood lipid levels did not significantly change, no severe side-effects observed.
Michelakis MD, et al. [21]	Prospective study administering dichloroacetate (DCA) to mitochondrial cell lines of patients diagnosed with GBM.	2	49	DCA depolarized mitochondria, increased mitochondrial reactive oxygen species, and induced apoptosis in GBM cells, as well as in putative GBM stem cells, both in vitro and in vivo. DCA sufficient to inhibit the target enzyme of DCA, pyruvate dehydrogenase kinase II, which was highly expressed in all glioblastomas.

## Conclusions

Preclinical studies have established that a ketogenic diet (KD) is correlated with improved prognosis in high-grade glioma mouse models. These findings were later applied to clinical studies, in which hyperglycemic individuals with GBMs generally had poorer survival rates than those with lower blood glucose levels. Recent clinical findings have demonstrated that induced hypoglycemia and a ketogenic diet are tolerable and can potentially be an adjuvant to standard treatments, such as surgery and chemoradiation. Other findings have advocated for KD as an malignant cell growth inhibitor and indicate that further studies including the ongoing clinical trials analyzing larger cohorts of GBM patients treated with a KD are needed to determine the breadth of impact a KD may have on GBM treatment.
